# Lactylation‐Related Gene *LILRB4* Predicts the Prognosis and Immunotherapy of Prostate Cancer Based on Machine Learning

**DOI:** 10.1111/jcmm.70669

**Published:** 2025-06-27

**Authors:** Qinghua Wang, Xin Qin, Yan Zhao, Wei Jiang, Mingming Xu, Xilei Li, Haopeng Li, Juan Zhou, Gang Wu

**Affiliations:** ^1^ Department of Urology Tongji Hospital, School of Medicine, Tongji University Shanghai China; ^2^ Department of ICU Tongji Hospital, School of Medicine, Tongji University Shanghai China

**Keywords:** lactylation, LILRB4, machine learning, prognosis, prostate cancer

## Abstract

Lactylation plays a pivotal role in the metabolic reprogramming, proliferation, migration and immune evasion of tumour cells. However, its specific impact on prostate cancer (PCa) remains poorly understood. This study aimed to investigate the role of lactylation related genes (LRGs) in PCa. LRGs were identified and analysed using data from The Cancer Genome Atlas (TCGA), DKFZ2018, GSE46602 and GSE70768 cohorts. Unsupervised clustering was employed to categorise patients with PCa into two distinct clusters. Prognostic models for PCa were developed using multiple machine learning techniques. LRGs signature was established and validated through training and validation sets. The role of leukocyte immunoglobulin‐like receptor B4 (LILRB4) in PCa was examined both in vitro and in vivo. Analysis of LRG expression and prognosis in patients with PCa revealed two distinct clusters with differing survival rates and immune responses. Machine learning models demonstrated the ability to predict survival risks, potentially aiding in the development of personalised treatment strategies. Additionally, LILRB4, a key LRG, promotes PCa progression by modulating the NF‐κB and PI3K/AKT pathways, highlighting its potential as a therapeutic target. LRGs exert a pivotal influence on PCa, impacting patient prognosis, immune response and drug sensitivity. The LRGs signature emerges as an essential prognostic tool and a promising therapeutic target for PCa.

## 1 Introduction

Prostate cancer (PCa) represents one of the most common malignancies in the male genitourinary system [1]. Recent epidemiological data indicate that PCa ranks as the second most frequently diagnosed cancer and the fifth leading cause of cancer‐related mortality among men worldwide [2]. In the United States, recent statistics indicate that PCa is now the most commonly diagnosed malignancy and the second leading cause of cancer mortality among men [3]. Projections for 2024 suggest that nearly 300,000 individuals will be diagnosed with PCa, with more than 35,000 succumbing to the disease [3]. In China, there has been a notable rise in PCa incidence in recent years [4]. The incidence is lower than that observed in Western developed countries, but a higher proportion of patients are diagnosed with advanced PCa in China [5].

Lactate, the final product of glycolysis, is produced during various physiological and pathological processes, including hypoxia, inflammation and cancer progression [6, 7]. However, recent research has demonstrated that lactate is not merely a metabolic by‐product; it also plays a key role in regulating numerous biological processes [8, 9]. In cancer cells, lactate levels are markedly elevated through the Warburg effect and lactate serves as the primary inducer of lactylation [10, 11, 12]. Lactylation, a form of post‐translational modification of proteins, has emerged as a significant factor in driving tumour progression and conferring chemoresistance across various cancers [13, 14]. This modification influences metabolic reprogramming, cellular proliferation, migration and immune evasion by directly or indirectly modifying histones or non‐histone proteins [15, 16, 17, 18]. Consequently, lactylation is increasingly recognised as a novel and potentially crucial therapeutic target in cancer treatment. In PCa, lactylation promotes tumour progression by modulating metabolic pathways, epigenetic regulation and tumour microenvironment remodelling. Lactate accumulation activates oncogenic signalling while inhibiting tumour‐suppressive pathways, thereby enhancing cellular proliferation, epithelial–mesenchymal transition (EMT) and metastasis. However, the role of lactylation, particularly in immune evasion and resistance to therapy, remains inadequately understood.

This study established a model focused on lactylation related genes (LRGs) by comparing PCa tissues with normal tissues, enabling precise prognostic predictions for PCa. Expression levels of 18 LRGs in patients with PCa were assessed, with all patients subsequently classified into two clusters based on their expression profiles. Patients in the high‐expression cluster exhibited poorer prognosis. A machine learning predictive model was then developed, incorporating three LRGs. This prognostic model demonstrated both stability and reliability, as validated across training and validation cohorts. Notably, the model effectively distinguished high‐ and low‐risk patient groups, enhancing prognosis prediction and offering insights into the immune landscape of PCa.

Leukocyte immunoglobulin‐like receptor B4 (LILRB4), a member of the LILR family located on 19q13.4, facilitates immune evasion in various cancers, including multiple myeloma, lung cancer and gastric cancer, by interacting with immune cells within the tumour microenvironment [19, 20, 21, 22]. However, the role of LILRB4 in PCa and its relationship with lactylation remain inadequately understood. Initial comparisons of lactylation levels and LILRB4 expression across various tissues revealed potential connections. Further investigations through cellular and animal models confirmed the link between lactylation and LILRB4, highlighting its critical role in PCa progression.

## 2 Materials and Methods

### 2.1 Data Retrieval and Cleaning

The TCGA cohort was sourced from The Cancer Genome Atlas Database (https://portal.gdc.cancer.gov), while the DKFZ2018 cohort was retrieved from cBioPortal (https://www.cbioportal.org/). The GSE46602 and GSE70768 cohorts were obtained from the Gene Expression Omnibus (http://www.ncbi.nlm.nih.gov/geo). The cohort comprising sequencing data and clinical information of Chinese patients with PCa, referred to as the Chinese PRAD cohort, was sourced from the Chinese Prostate Cancer Genome and Epigenome Atlas (https://ngdc.cncb.ac.cn/bioproject/browse/PRJCA001124). For microarray data, expression data were processed using the limma software package for background correction and log2 transformation [23]. The ‘Normalize Between Arrays’ function was utilised to standardise the microarray data and eliminate systematic biases. High‐throughput RNA‐seq data were converted into transcripts per million (TPM) format, allowing for direct comparison with microarray data [24]. Clinical data cleaning involved excluding patients with a survival time of 0. Twenty‐two LRGs (Table S1) identified from prior studies were included to ensure research consistency [25, 26].

### 2.2 Differential Expression Analysis

Expression analysis was performed using the DESeq2 R software package [27]. Genes showing a significant fold change > 1 and a *p* < 0.05 were considered differentially expressed.

### 2.3 Survival Analysis

Survival analysis was conducted using the survival R software package. Continuous variables were categorised based on optimal cut‐off values, determined using the survminer R software package.

### 2.4 Consensus Clustering Analysis

Unsupervised clustering of the lactylation gene expression matrix from TCGA patients was conducted using the ConsensusClusterPlus R software package [28], employing 50 iterations and resampling 80% of the samples. The Euclidean distance method was used for distance calculations, while the partitioning around medoids (PAM) method was applied to determine the optimal number of clusters.

### 2.5 Immune Infiltration Analysis

The ESTIMATE (Estimation of Stromal and Immune cells in Malignant Tumour tissues using Expression data) algorithm was used to assess tumour purity and immune cell infiltration. The ESTIMATE, stromal and immune scores were calculated using the ESTIMATE R software package [29]. Additionally, marker gene sets for 28 immune cell types were derived from existing literature and the single‐sample gene set enrichment analysis (ssGSEA) algorithm was employed to evaluate immune cell infiltration across different patient groups [30].

### 2.6 GSVA

Gene set variation analysis (GSVA) is a non‐parametric, unsupervised method for gene set enrichment analysis [31], primarily used to assess variations in gene set enrichment across samples. It enables the identification of differential activity in biological pathways or gene sets under different conditions. The immune‐related gene set c7.immunesigdb.v2024.1.Hs.symbols.gmt utilised in this study was obtained from the Molecular Signature Database (http://software.broadinstitute.org/gsea/msigdb). GSVA was implemented using the GSVA R software package.

### 2.7 WGCNA

Weighted gene co‐expression network analysis (WGCNA) was performed using the WGCNA R software package [32] to identify differentially expressed genes across various groups following clustering and immune‐related gene module analysis. The co‐expression network was constructed using TCGA data, with outlier samples excluded. Pairwise gene correlations were computed to form an adjacency matrix. An optimal soft‐thresholding parameter (*β*) was chosen to accentuate strong gene associations while diminishing weaker ones, ensuring the network exhibited scale‐free properties, aligning with biological realities. The topological overlap matrix (TOM), derived from the adjacency matrix, was calculated to better represent gene network relationships. The dynamic tree cut algorithm was applied to construct a gene dendrogram based on TOM similarity. A suitable cutting height was determined to partition the dendrogram into distinct modules. Correlations between each gene module and sample phenotypes were then calculated. For key modules, genes with a gene significance (GS) value > 0.6 and a module membership (MM) value > 0.8 were selected as key genes for further analysis.

### 2.8 Machine Learning for Prognostic Model Construction

To develop the optimal prognostic model, a comprehensive approach incorporating 10 machine learning algorithms was employed. These included CoxBoost, Lasso, Ridge, Enet, StepCox, survival‐SVM, GBM, plsRcox, RSF and SuperPC. Initially, variable selection was performed using algorithms with built‐in selection functions, such as StepCox, Lasso, CoxBoost and RSF. Subsequently, the selected variables were input into model‐building algorithms for model fitting. CoxBoost analysis was carried out using the CoxBoost R software package, with 10‐fold cross‐validation applied to identify the optimal number of boosting steps. The glmnet R package was utilised for constructing Lasso, Ridge and Enet models, with 10‐fold cross‐validation determining the optimal regularisation parameter (lambda) [33]. The StepCox model was fitted using the survival R software package, while the survival‐SVM model was built with the survivalsvm R software package. The GBM model was constructed using the gbm R package, also incorporating 10‐fold cross‐validation. The plsRcox model was fitted using the plsRcox R package. For the RSF model, the rfsrc function within the randomForestSRC R software package was employed, with the ntree parameter set to 1000. Finally, the SuperPC model was built using the superpc R software package and 10‐fold cross‐validation was used to determine the best‐fitting model.

### 2.9 GSEA

Gene set enrichment analysis (GSEA) was performed using the clusterProfiler R package [34], with species annotation provided by the org.hs.eg package. The reference gene sets were sourced from the gene ontology (GO) and Kyoto Encyclopedia of Genes and Genomes (KEGG) databases. To ensure robust analysis, the minimum number of genes was set to 10 and the maximum was limited to 500. A false discovery rate (FDR) threshold of < 0.05 was applied as the criterion for significant enrichment, with gene sets meeting this threshold deemed to have biologically meaningful enrichment patterns.

### 2.10 Prediction of Tumour Immunotherapy Response and Drug Sensitivity

Tumour Immune Dysfunction and Exclusion (TIDE) is an algorithm designed to predict the responsiveness of cancer individuals to immune checkpoint inhibitors, specifically PD‐1/PD‐L1 inhibitors [35]. After normalising the gene expression data, the TIDE score, dysfunction score and exclusion score were calculated for each patient using the TIDE database (http://tide.dfci.harvard.edu/). Drug sensitivity predictions were made using the oncoPredict package [36]. The standard expression matrix of GDSC2 and the corresponding IC50 values for each cell line and drug were retrieved from GitHub (https://github.com/maese005/oncoPredict/tree/main/vignettes), which served as the training set for subsequent analyses [37]. This approach enabled us to gain insights into both immune drug sensitivity and microenvironment characteristics relevant to cancer treatment.

### 2.11 Construction of Nomogram

Univariate and multivariate Cox regression analyses were performed using the survival R software package. To identify the most appropriate model, the Akaike information criterion (AIC) was employed [38]. For the derived variables, the rms R package was utilised to generate nomograms and calibration curves. The pec R package was used to construct time‐dependent area under the curve (AUC). To evaluate the practical utility of the prediction model in clinical decision making, the ggDCA R software package was applied to compare net benefits across different decision‐making strategies within a specified threshold interval.

## 3 Patient Samples and Cell Culture

Tissue samples from all patients were obtained from Tongji Hospital, Tongji University, between 2022 and 2024 (Table S2). The PC3 and DU145 cell lines were sourced from the Cell Bank of the Chinese Academy of Sciences (Shanghai, China). Lactate was purchased from MCE (China).

### 3.1 Western Blot

Cell proteins were extracted from cell lysates and tissue homogenates, with nuclear and cytoplasmic protein extraction performed using a Beyotime kit (China). Denatured proteins were separated based on size through 10% SDS‐PAGE and transferred onto a PVDF membrane activated by methanol using the rapid transfer buffer (Beyotime, China) at 200 mA. The membrane was blocked to prevent non‐specific antibody binding and incubated overnight with a primary antibody. The following day, a secondary antibody was applied and bands were detected using enhanced chemiluminescence (Beyotime, China) and imaged using a gel documentation system. The results were analysed using ImageJ software.

### 3.2 Cell Transfection

To establish the LILRB4 overexpression and knockdown cell models, LILRB4 plasmid (Youze, China) and short hairpin RNA (shRNA) constructs (Youze, China) were transfected using Lipofectamine 2000 (Invitrogen, USA). The sequences of the shRNA constructs were shLILRB4#1: GCTCATAGTCTCAGGATCCTT and shLILRB4#2: CTCGGGAGTACCGTCTGGATA. The efficiency of transfection was verified via western blot analysis.

### 3.3 CCK‐8 Assay

Cell viability was assessed using the CCK‐8 assay. Approximately 5000 cells were seeded into 96‐well plates and gently agitated for even distribution. On subsequent days, the medium was replaced with freshly prepared medium containing 10% CCK‐8 reagent (Beyotime, China). The plate was incubated for 1 h at 37°C and the optical density (OD) was measured using a microplate reader.

### 3.4 Colony Formation Assay

PCa cells (500 cells/well) were seeded in six‐well plates and cultured for approximately 2 weeks, with medium changes every 4 days. Once colonies formed, the cells were fixed and stained.

### 3.5 EdU Assay

Cells were seeded in 96‐well plates at a suitable density and cultured overnight to allow attachment. The BeyoClick EdU Cell Proliferation Kit with Alexa Fluor 555 (Beyotime, China) was used to assess cell proliferation. The proliferation rate was determined by calculating the ratio of EdU‐positive cells to Hoechst 33342‐stained cells.

### 3.6 Wound Healing Assay

PC3 and DU145 cells were grown to approximately 90% confluence and then scratched using a 200 μL sterile pipette. The cells were cultured in a medium with 2% FBS and images were captured at 0 and 24 h using an inverted microscope.

### 3.7 Transwell Assay

In the Transwell invasion assay, cells were seeded in the upper chamber pre‐coated with Matrigel (Corning, USA) and a medium containing 20% FBS was added to the lower chamber. After 48 h, non‐invading cells on the upper surface of the membrane were carefully removed and the invading cells were fixed, stained and air‐dried. The number of invaded cells was quantified using a microscope. A similar Transwell migration assay was conducted without Matrigel.

### 3.8 Immunofluorescence (IF)

PCa cells were seeded onto slides and incubated for 24 h. Cells were fixed with 4% paraformaldehyde, followed by permeabilisation with 0.5% Triton‐X100 (Beyotime, China). After blocking with 5% BSA for 1 h, cells were incubated overnight with the primary antibody. The following day, cells were treated with a fluorescent secondary antibody (Abclonal, China) in a light‐protected environment. The final step involved staining with an anti‐fade agent containing DAPI (Solarbio, China). Observations were made using a fluorescence microscope and images were captured for documentation.

### 3.9 Haematoxylin and Eosin (HE)

Tissue sections were first cleared of paraffin wax using xylene and then progressively passed through decreasing concentrations of alcohol to water for tissue hydration. Haematoxylin was applied to stain the nuclei blue, followed by washing to remove excess stain. Eosin was then applied to stain the cytoplasm pink. To complete the process, increasing concentrations of alcohol were used to dehydrate the tissue and xylene was used to clear the tissue before imaging.

### 3.10 Immunohistochemistry (IHC)

Tissue samples underwent fixation, dehydration, clearing and embedding. Sections of 5 μm thickness were mounted, dewaxed and rehydrated before antigen retrieval. Endogenous peroxidase activity was blocked and non‐specific binding was minimised using serum albumin. The sections were then incubated with a primary antibody, followed by a biotinylated secondary antibody. DAB substrate development was used to visualise the antigen–antibody reaction and slides were counterstained with haematoxylin. Dehydration, clearing and mounting were performed and the slides were examined microscopically, with brown staining indicating target cell localisation, captured digitally.

### 3.11 Xenograft Assay

Male BALB/c nude mice, approximately 4 weeks old, were purchased from Shanghai Shengchang Co. Ltd. (Shanghai, China). The mice were randomly assigned to three groups: the sh‐NC group, the sh‐LILRB4#1 group and the sh‐LILRB4#2 group. After 3 days of acclimatisation under specific pathogen‐free conditions, the mice underwent the experimental procedure. Each mouse was injected with 1 × 10^6^ PC3 cells in 100 μL of RPMI‐1640 medium into the forelimb axilla. Tumour measurements were taken every 5 days. After approximately 30 days, all tumours were excised for weight measurement and IHC analysis.

### 3.12 Statistical Analysis

Statistical analyses and visualisations were performed using R (version 4.41), SPSS Statistics 26 and GraphPad Prism 10 software. *T* tests or non‐parametric tests were used for inter‐group comparisons based on normality testing. For comparisons of two or more groups, one‐way ANOVA or the Kruskal–Wallis rank sum test was applied. Bilateral Fisher's exact test was used for contingency tables and Pearson's or Spearman's correlation tests were used for correlation analysis between variables; *p < 0.05 was considered statistically significant. All data are presented as mean ± SD.

## 4 Results

### 4.1 LRGs Exhibit Different Expression Patterns and Predict the Poor Prognosis of PCa

**FIGURE 1:**
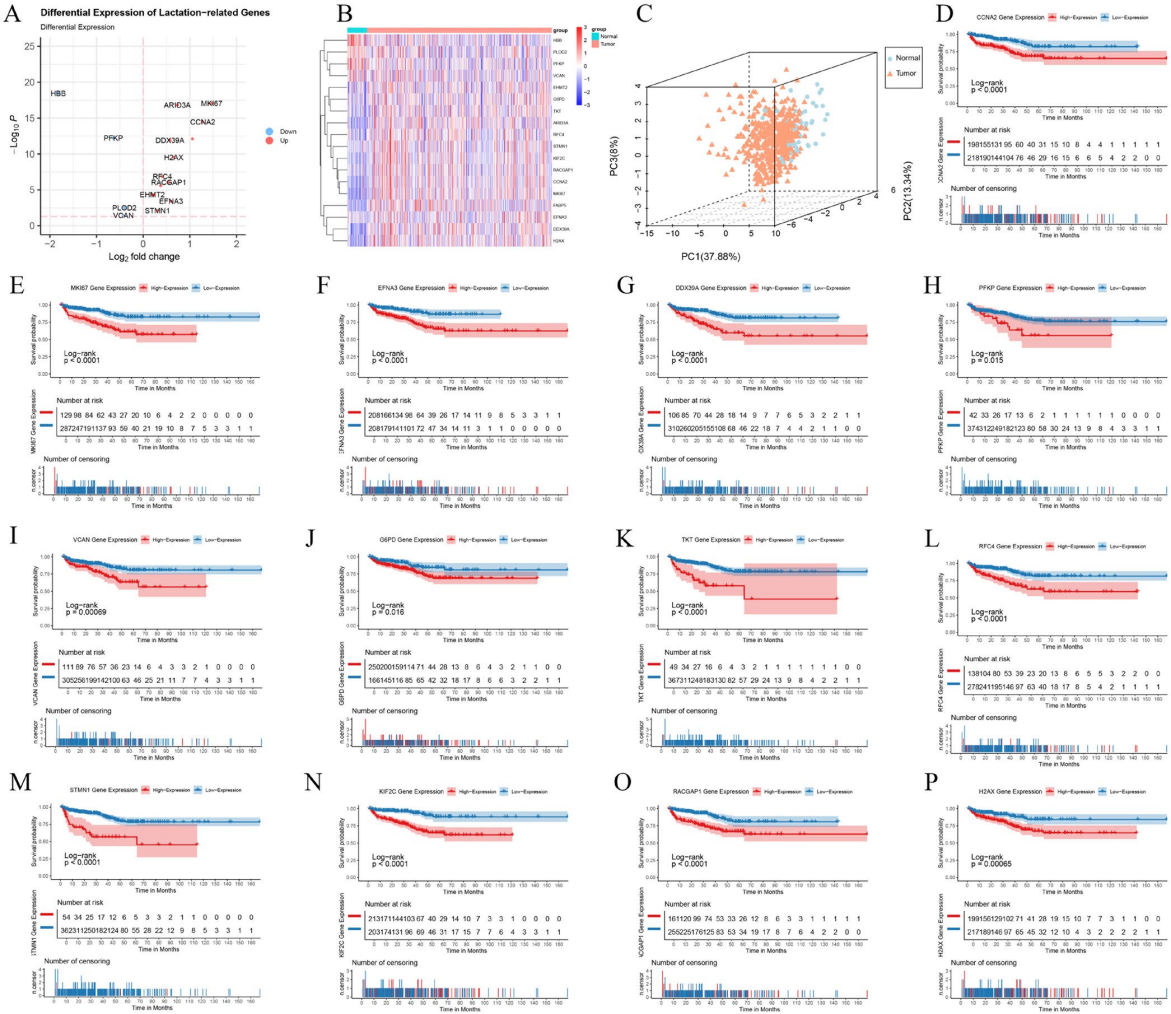
Differential expression and prognostic analysis of LRGs in PCa. (A) The volcano plot illustrated the differentially expressed LRGs in PCa and the control group. (B) The heatmap showed the expression profiles of the differentially expressed LRGs. (C) The three‐dimensional scatter plot demonstrated the PCA results of the LRGs. (D–P) The Kaplan–Meier curves displayed the affect of the LRGs expression on the prognosis of PCa.

To elucidate the relationship between lactylation and PCa, differential expression analysis of LRGs was performed using the TCGA cohort with the DESeq2 R software package. The analysis identified 18 differentially expressed LRGs in PCa (Figure 1A,B). Specifically, HBB, PLOD2, PFKP and VCAN were down‐regulated, while genes such as *EHMT2* were up‐regulated in PCa. Principal component analysis (PCA) revealed distinct lactylation gene expression patterns between patients with PCa and the control group (Figure 1C). Additionally, the impact of these lactylation genes on biochemical recurrence‐free survival (BCRFS) in patients with PCa was explored. Survival analysis revealed that 13 of the 18 differentially expressed LRGs had a significant influence on prognosis (Figure 1D–P), with high expression of these genes associated with a negative prognosis.

### 4.2 Clustering Based on LRGs Presents Distinct Tumour Immune Microenvironment Landscapes

**FIGURE 2:**
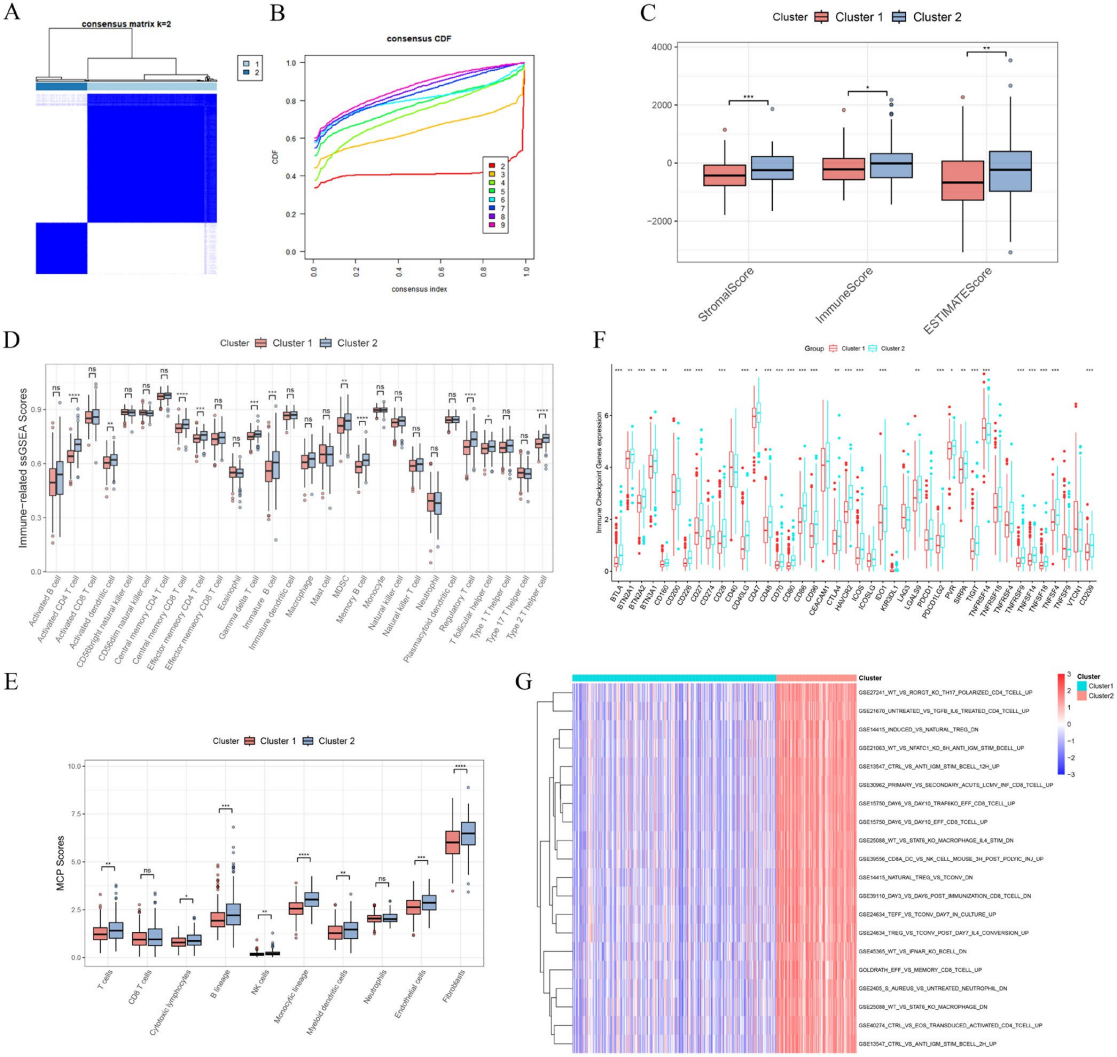
Unsupervised clustering analysis based on LRGs suggested different immune microenvironment landscapes. (A, B) Patients were categorised into two distinct groups by selecting *k* = 2 via the unsupervised clustering method. (C) The differences in immune, stromal and ESTIMATE scores among patients in the two groups of consensus clustering. (D) The ssGSEA scores of 28 immune cells among the two groups. (E) The infiltration levels of 10 different immune cells obtained by the MCP counter algorithm. (F) The analysis of immune checkpoint genes in the two clusters. (G) A heatmap of the differences in the activity of immune‐related gene sets obtained by GSVA. *p < 0.05, **p < 0.01, ***p < 0.001, ****p < 0.0001.

Next, the ConsensusClusterPlus R software package was utilised to identify potential sub‐types or clustering patterns within the lactylation gene expression profiles of patients with PCa. Based on the optimal *k* value derived using the PAM method, the clustering structure at *k* = 2 showed high stability, with the CDF curve levelling off at this value (Figure 2A,B). Consequently, the patients were divided into two clusters. Given lactylation's potential influence on the immune microenvironment and its role in cancer progression [14], the differences in immune microenvironment landscapes between the two clusters were further examined. The ESTIMATE algorithm was used to calculate immune, stromal and ESTIMATE scores for both clusters (Figure 2C). Cluster 1 displayed significantly lower immune scores compared to cluster 2. Immune cell infiltration analysis revealed that the abundance of immune cell infiltration in cluster 1 was substantially lower than in cluster 2 (Figure 2D). Among the 28 immune cell types analysed, 11 showed significant differences, with higher immune cell infiltration observed in cluster 2. Notably, CD4^+^ T cells, central memory CD8^+^ T cells and effector memory CD4^+^ T cells were significantly enriched in cluster 2, suggesting that cluster 2 may have a more robust and sustained anti‐tumour immune response within the PCa microenvironment. B cells are a crucial component of humoral immunity [39]. The memory B cell and immature B cell were also significantly enriched in cluster 2. To further assess immune cell infiltration levels, the MCP counter tool was used, indicating lower immune cell infiltration in cluster 1 (Figure 2E). Moreover, the expression levels of many immune checkpoint molecules differed significantly between the two clusters, underscoring the potential role of lactylation in modulating immunotherapy strategies (Figure 2F). Immune‐related gene sets were obtained from MsigDB and GSVA was used to evaluate the enrichment of these gene sets in the two clusters. The results demonstrated that most immune‐related gene sets with significant differences were enriched in cluster 2, indicating that immune‐related pathways were more active in this cluster compared to cluster 1 (Figure 2G).

### 4.3 Identification of the Most Immune‐Relevant Module Based on WGCNA

**FIGURE 3:**
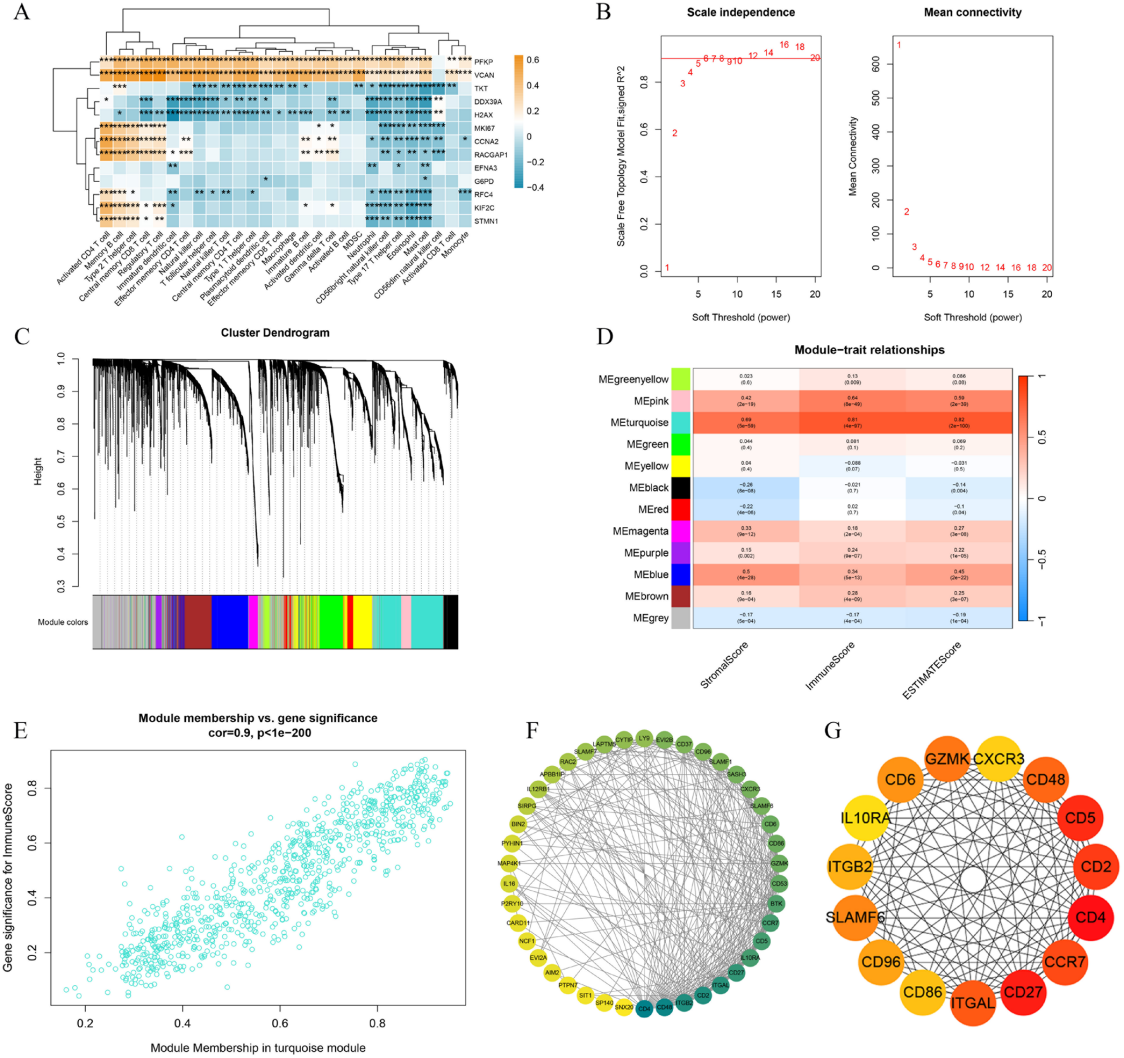
Identification of immune‐related modules by WGCNA. (A) The correlation heatmap presented the relationship between LRGs and the ssGSEA scores of 28 immune cell types. (B) The mean connectivity as a function of the scale‐free fit index and the soft‐thresholding power for various soft‐thresholding β values. (C) The clustering dendrogram of the co‐expression network modules in accordance with the 1‐TOM matrix. (D) The correlation heatmap between genes in each module and the immune score. (E) The correlation scatter plot demonstrating the module eigengenes of the turquoise module. (F) Protein–protein interaction information from the STRING database (https://cn.string‐db.org/). (G) Protein–protein interaction information from the Cytoscape software to enhance the network quality. *p < 0.05, **p < 0.01, ***p < 0.001, ****p < 0.0001.

To explore the underlying causes of the distinct immune microenvironments identified through lactylation gene clustering analysis, expression analysis was performed on patients from the two clusters (Figure 3A). Genes with an absolute fold change > 0.5 and a *p* < 0.05 were considered differentially expressed, resulting in 6476 such genes. These differential genes were then incorporated into WGCNA to identify the key modules most relevant to immunity.

Following data standardisation and pre‐processing of the TCGA dataset, the network topology was analysed (Figure 3B,C). A soft threshold of six was selected to ensure that the constructed gene co‐expression network exhibited scale‐free properties, with a scale‐free *R* ^2^ value of 0.9. A cut‐off of 0.25 and a minimum module size of 30 were applied to identify gene modules. Pearson's correlation analysis was then performed to examine the relationship between module eigengenes and immune‐related scores (Figure 3D,E). The turquoise module showed the highest correlation with immune‐related scores in PCa (*r* = 0.81, *p* = 4e‐97), making it the key module selected for further investigation.

Key genes within the turquoise module were identified based on MM values > 0.6 and GS values exceeding 0.8. To reveal the association patterns among key module proteins and identify critical nodal proteins, protein–protein interaction (PPI) data were integrated from the STRING database in this study (Figure 3F). The protein interaction data were then imported into Cytoscape to enhance network visualisation (Figure 3G). The Cytohubba plugin in Cytoscape, along with the MCC algorithm, was applied to identify hub genes with critical regulatory roles in the network.

### 4.4 Construction of PCa Prognostic Prediction Models Using Multiple Machine Learning Approaches

Univariate Cox regression analysis was conducted on the key genes identified from the WGCNA key module, selecting genes with a *p* < 0.01 for further analysis. The TCGA cohort was used as the training set, while the Chinese PRAD, DKFZ2018, GSE46602 and GSE70768 cohorts served as validation sets. A variety of machine learning algorithms, including random survival forests (RSF) and CoxBoost, were applied to construct prognostic models. The concordance index (C‐index) was employed to assess the consistency between the model's predicted survival time and the actual survival time.

**FIGURE 4:**
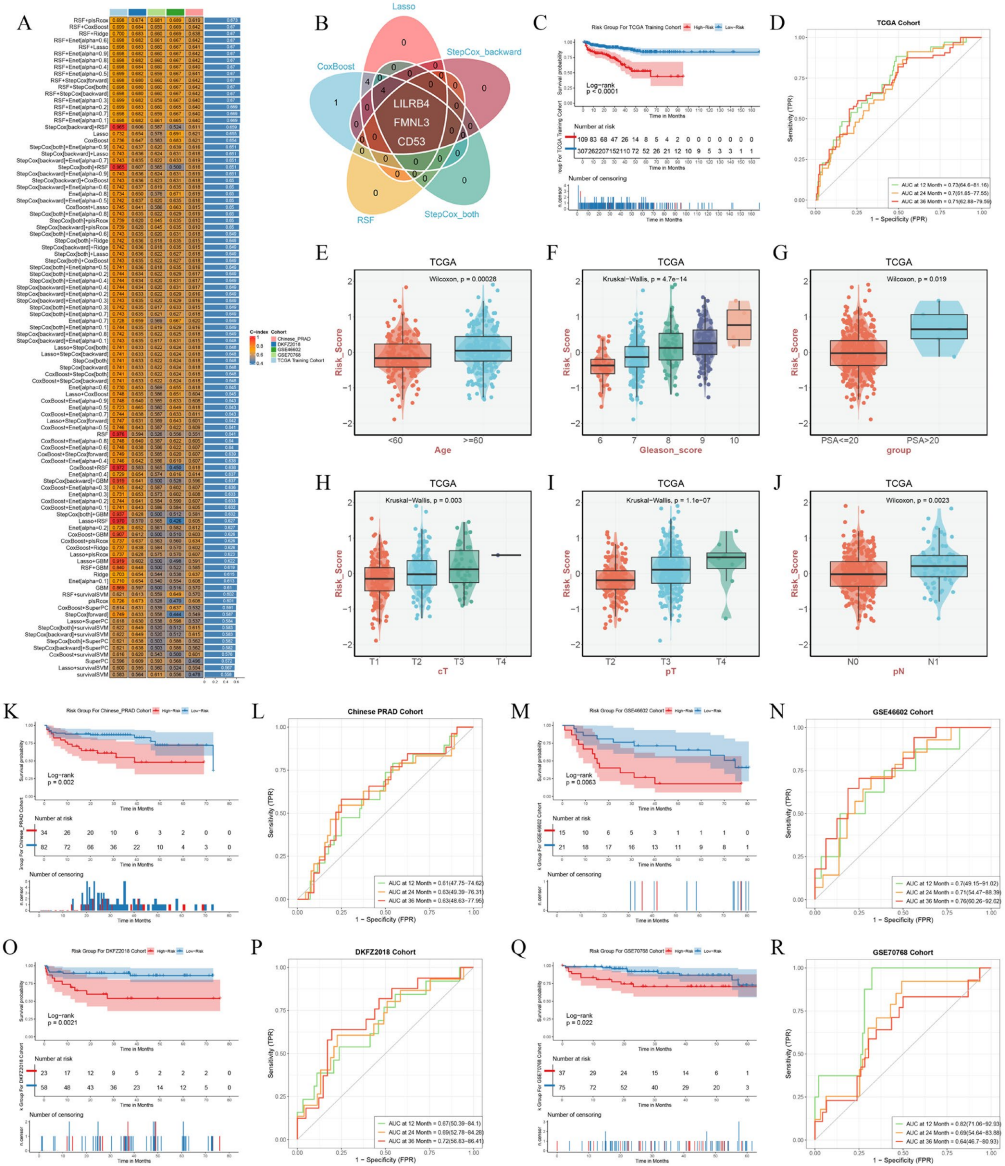
Construction of prognostic prediction models for PCa based on multiple machine learning algorithms. (A) The C‐index of each machine learning algorithm in the training set and multiple validation sets. (B) The Venn diagram about the variables jointly selected by five machine learning algorithms with variable screening functions. (C) The Kaplan–Meier (KM) curve of the TCGA training set and the (D) ROC curves. The risk scores among patients with different age (E), Gleason score (F), PSA level (G), cT (H), pT (I) and pN stage (J). The KM and ROC curves using risk scores in the Chinese PRAD cohort (K, L), GSE46602 cohort (M, N), DKFZ2018 cohort (O, P) and GSE70768 cohort (Q, R).

The C‐index heatmap revealed that the combination of RSF and plsRcox yielded the highest average C‐index of 0.673 (Figure 4A). Five machine learning algorithms with variable selection capabilities all identified LILRB4, FMNL3 and CD53, suggesting a strong association between these genes and the prognosis of PCa (Figure 4B).

Using the combined RSF and plsRcox models, risk scores for the training and validation sets were calculated. The optimal cut‐off value, corresponding to the point closest to the upper left corner of the receiver operating characteristic (ROC) curve, was selected to classify patients into high‐ and low‐risk groups. In the training set, the high‐risk group exhibited significantly poorer prognosis compared to the low‐risk group, with AUC values for predicting 1‐, 2‐ and 3‐year survival of 0.73, 0.7 and 0.71 respectively. Additionally, PCa individuals with advanced age, high Gleason score, elevated PSA levels and later grade stages had significantly higher risk scores, indicating that the risk score serves as an unfavourable prognostic factor (Figure 4E–J). In all validation sets, patient prognosis aligned with the training set results (Figure 4K–R).

### 4.5 Construction of Prognostic Nomogram, Prediction of Immunotherapy and Drug Sensitivity in Different Risk Groups

**FIGURE 5:**
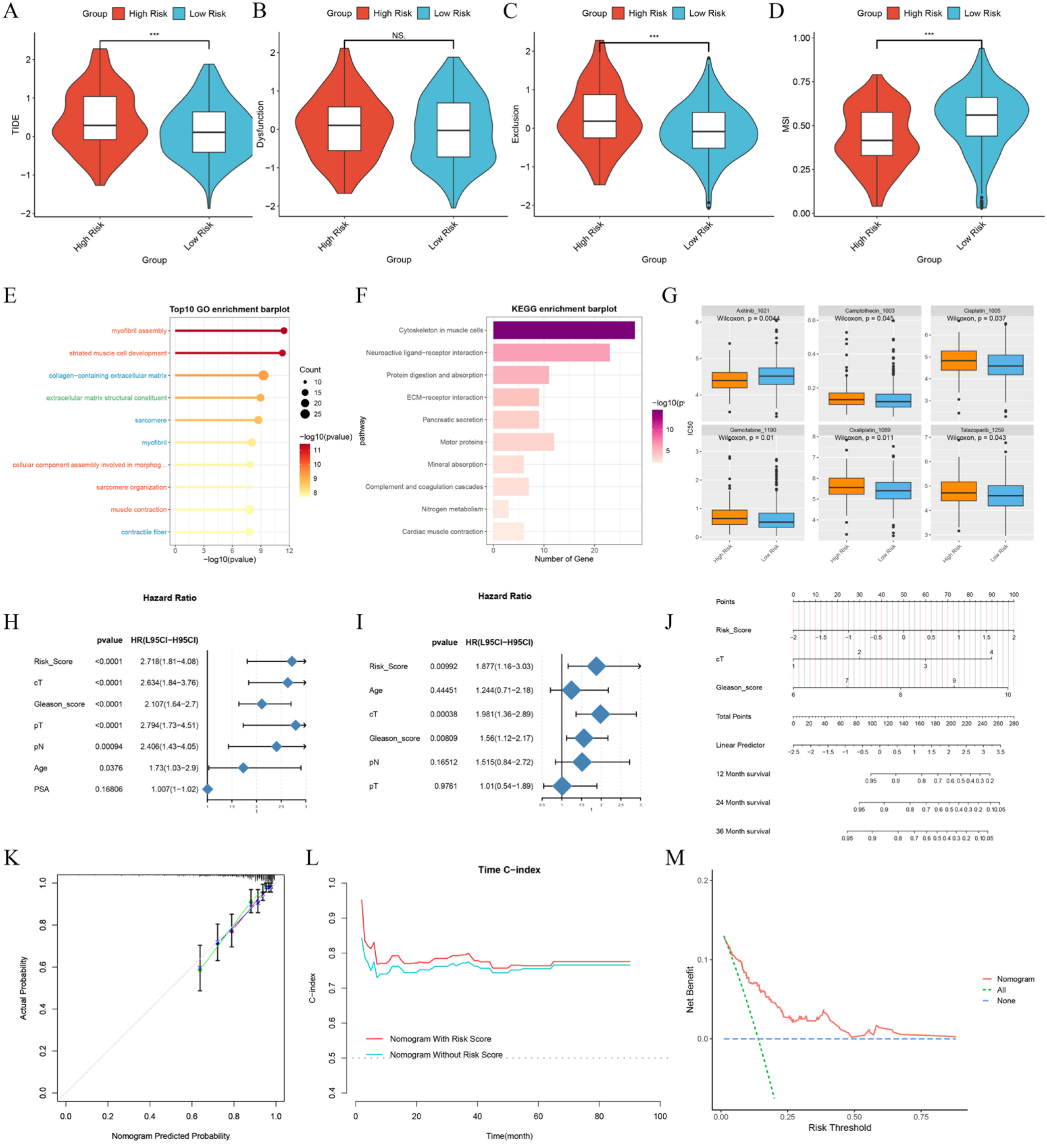
Immune therapy, drug sensitivity prediction and nomogram construction for patients. (A–D) Violin plots displayed the TIDE score, exhaustion score, escape score and MSI level of patients in different groups. Enrichment analysis of differentially expressed genes between the high‐ and low‐risk groups by relying on the GO database (E) and KEGG database (F). (G) The IC50 values of several chemotherapy drugs in patients. (H, I) Forest plots showed the results of univariate and multivariate COX regression for risk score, Gleason score, cT, pT, pN, age and PSA level. (J) Nomogram for predicting BCRFS prognosis of PCa patients. (K) Calibration curve of the nomogram. (L) Time‐dependent C‐index curves of the nomogram with and without the inclusion of risk score. (M) Clinical decision curve of the nomogram. ***p < 0.001.

The TIDE score, T‐cell exhaustion score, escape score and microsatellite instability (MSI) level for each patient in the TCGA cohort were retrieved from the TIDE database. High‐risk patients exhibited elevated TIDE and escape scores, indicating that tumour cells in these individuals were more likely to evade immune surveillance, promoting proliferation and metastasis and predicting poorer responses to immunotherapy (Figure 5A–D). Conversely, the low‐risk group displayed higher MSI, suggesting a greater mutational burden and potentially enhanced immune responses. GO enrichment analysis identified significant enrichment in pathways related to muscle fibre, striated muscle cell development and muscle contraction (Figure 5E). KEGG enrichment analysis revealed significant associations with muscle cell and neuroactive ligand–receptor interaction pathways (Figure 5F).

Drug sensitivity predictions indicated that high‐risk patients were more responsive to axitinib, while low‐risk patients showed increased sensitivity to camptothecin, cisplatin, gemcitabine, oxaliplatin and talazoparib (Figure 5G). These results suggest that the model's risk score could be instrumental in guiding personalised immunotherapy and chemotherapy treatment decisions.

A nomogram was developed to accurately calculate the likelihood of specific disease events, such as recurrence, metastasis or survival time, by integrating multiple individualised patient characteristics. This approach facilitates the implementation of more aggressive adjuvant treatment strategies for high‐risk patients. Univariate and multivariate Cox regression analyses (Figure 5H,I) identified the risk score, clinical T stage (cT), Gleason score, pathological T stage (pT), pathological N stage (pN) and age as significant adverse prognostic factors for BCRFS in patients with PCa.

The prognostic nomogram for PCa was constructed using the risk score, cT and Gleason score, achieving a C‐index of 0.778 (Figure 5J). The calibration curve indicated close alignment between predicted and actual probabilities, confirming the accuracy of the nomogram's predictions (Figure 5K). The time‐dependent C‐curve demonstrated that the nomogram, which included the risk score, outperformed the version without it, with a C‐index around 0.78 for predicting survival at various time points, underscoring its robust prognostic performance (Figure 5L). Clinical decision curve analysis revealed that, across most threshold ranges, the clinical net benefit of the nomogram exceeded that of no intervention, suggesting that this nomogram could serve as a valuable clinical tool for predicting patient outcomes and guiding more personalised treatment strategies (Figure 5M).

### 4.6 The LILRB4 and Lactylation Levels Are Elevated in PCa

**FIGURE 6:**
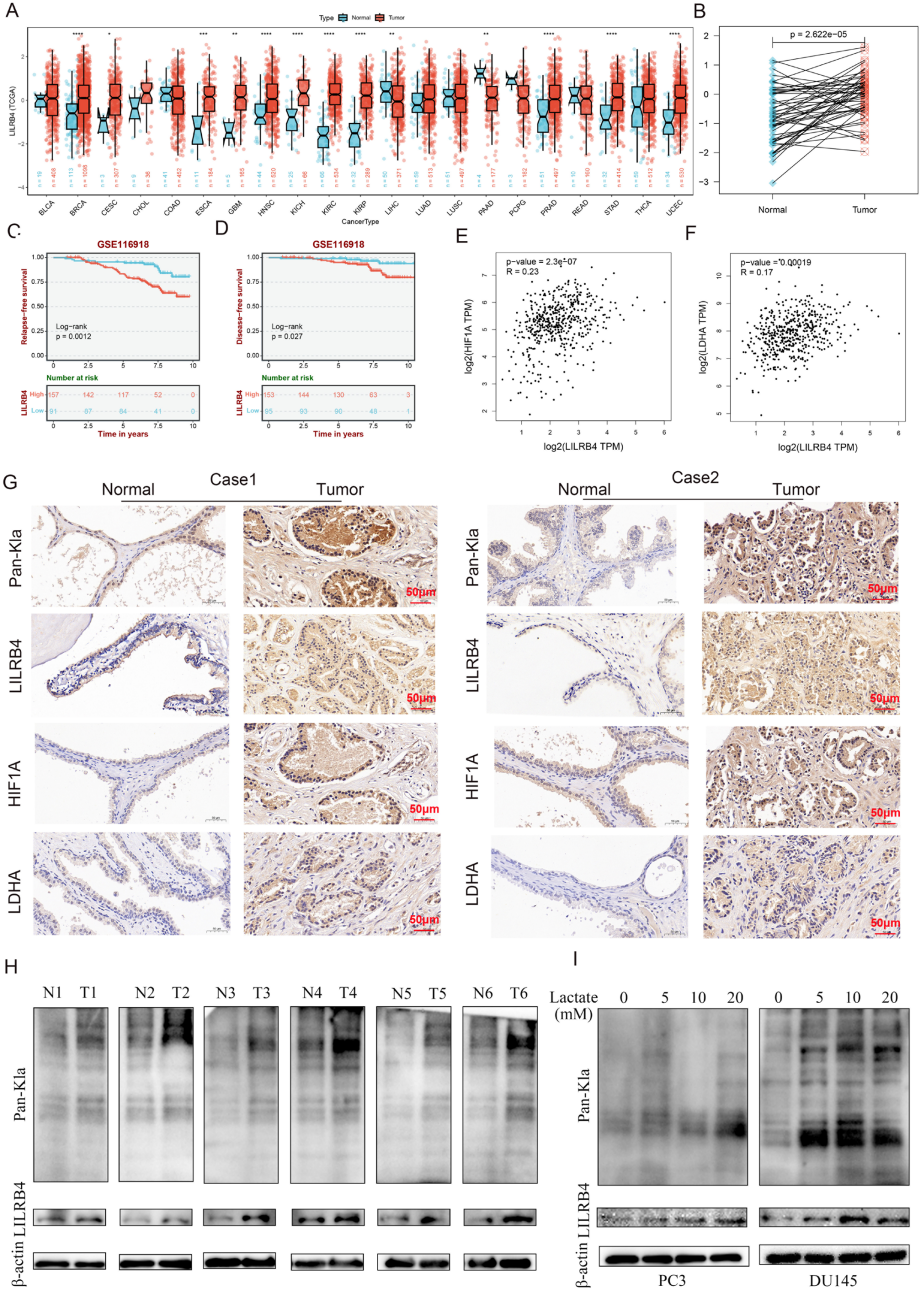
Lactate‐mediated LILRB4 is up‐regulated in PCa. (A) The pan‐cancer analysis of LILRB4 in Sparkle database (https://grswsci.top/). (B) The analysis of LILRB4 in paired PCa tissues of Sparkle database. The relapse‐free survival (C) and disease‐free survival (D) of LILRB4 in GSE116918 (https://rookieutopia.hiplot.com.cn/app_direct/BEST/). The relationship between LILRB4 and HIF1A (E), LDHA (F) from GEPIA (http://gepia2.cancer‐pku.cn/#index). (G) IHC showed the expression levlels of Pan‐Kla, LILRB4, HIF1A and LDHA. (H) The results of western blot demonstrated the levels of Pan‐Kla and LILRB4. (I) The LILRB4 expression was measured by western blot after treatment with lactate (0, 5, 10 and 20 mM) for 48 h. *p < 0.05, **p < 0.01, ***p < 0.001, ****p < 0.0001.

Among the three machine learning associated genes, *LILRB4*, *FMNL3* and *CD53*, only *LILRB4* was up‐regulated. Analysis using the BEST database revealed a significant increase in *LILRB4* expression across various tumours, particularly in PCa, with its expression also elevated in paired PCa tissue samples (Figure 6A,B). Patients with higher *LILRB4* expression exhibited poorer relapse‐free survival (RFS) and disease‐free survival (DFS) (Figure 6C,D). Previous studies have established that LDHA and HIF1A are closely related to lactate metabolism [40, 41]. A positive correlation was observed between *LILRB4* expression and both proteins (Figure 6E,F). To explore the relationship between *LILRB4* and lactylation, this study analysed the expression of *LILRB4* alongside these proteins. IHC staining revealed that *LILRB4*, pan‐lactylation and glycolysis‐related proteins (LDHA, HIF1A) were significantly up‐regulated in PCa tissues (Figure 6G). This was further confirmed by Western blot analysis, which showed elevated levels of both pan‐lactylation and LILRB4 in PCa tissues (Figure 6H). In PCa cells treated with varying concentrations of lactate under normoxic conditions for 48 h, expression levels of pan‐lactylation and LILRB4 increased in a concentration dependent manner (Figure 6I), while LILRB4 mRNA levels were up‐regulated exclusively in the 20 mM treatment group (Figure S1). These results suggest that lactate may mediate LILRB4 expression in PCa and that lactylation is likely involved in PCa tumorigenesis.

### 4.7 Lactate‐Mediated LILRB4 Promotes PCa Cells Proliferation In Vitro

**FIGURE 7:**
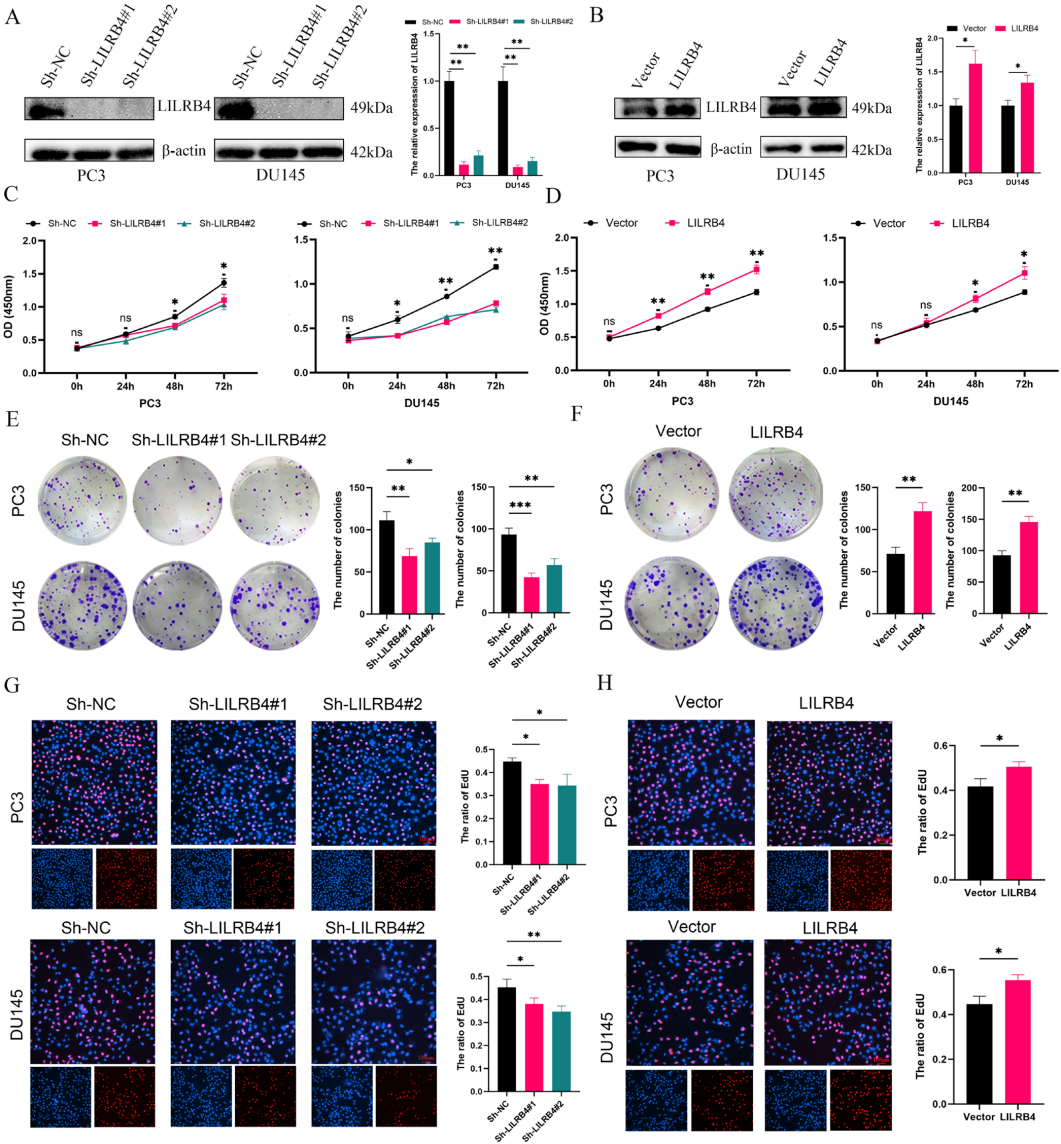
LILRB4 promotes PCa cells proliferation in vitro. (A) PC3 and DU145 cells were transfected with Sh‐LILRB4. (B) The overexpression models were constructed in PC3 and DU145. CCK8 assay was used to detect the cell viability after LILRB4 silencing (C) and up‐regulation (D). Tumour cells growth of PC3 and DU145 after LILRB4 silencing (E) and up‐regulation (F) was evaluated by colony formation assay. The EdU assay demonstrated the proliferation rate of PC3 and DU145 cells after LILRB4 silencing (G) and up‐regulation (H). *p < 0.05, **p < 0.01, ***p < 0.001.

To investigate the functional role of LILRB4 in PCa, LILRB4 down‐regulation and up‐regulation models were established using PC3 and DU145 cell lines (Figure 7A,B). Western blot analysis confirmed the transfection efficiency. CCK‐8 assays demonstrated that LILRB4 knockdown reduced cell viability, whereas overexpression of LILRB4 enhanced cell proliferation in both PC3 and DU145 cells (Figure 7C,D). The colony formation assay further showed that PCa cells with high LILRB4 expression had a greater ability to form colonies (Figure 7E,F). Additionally, the EdU assay confirmed an increased cell proliferation rate upon LILRB4 up‐regulation (Figure 7G,H).

### 4.8 LILRB4 Facilitates PCa Cell Metastasis and EMT Process

**FIGURE 8:**
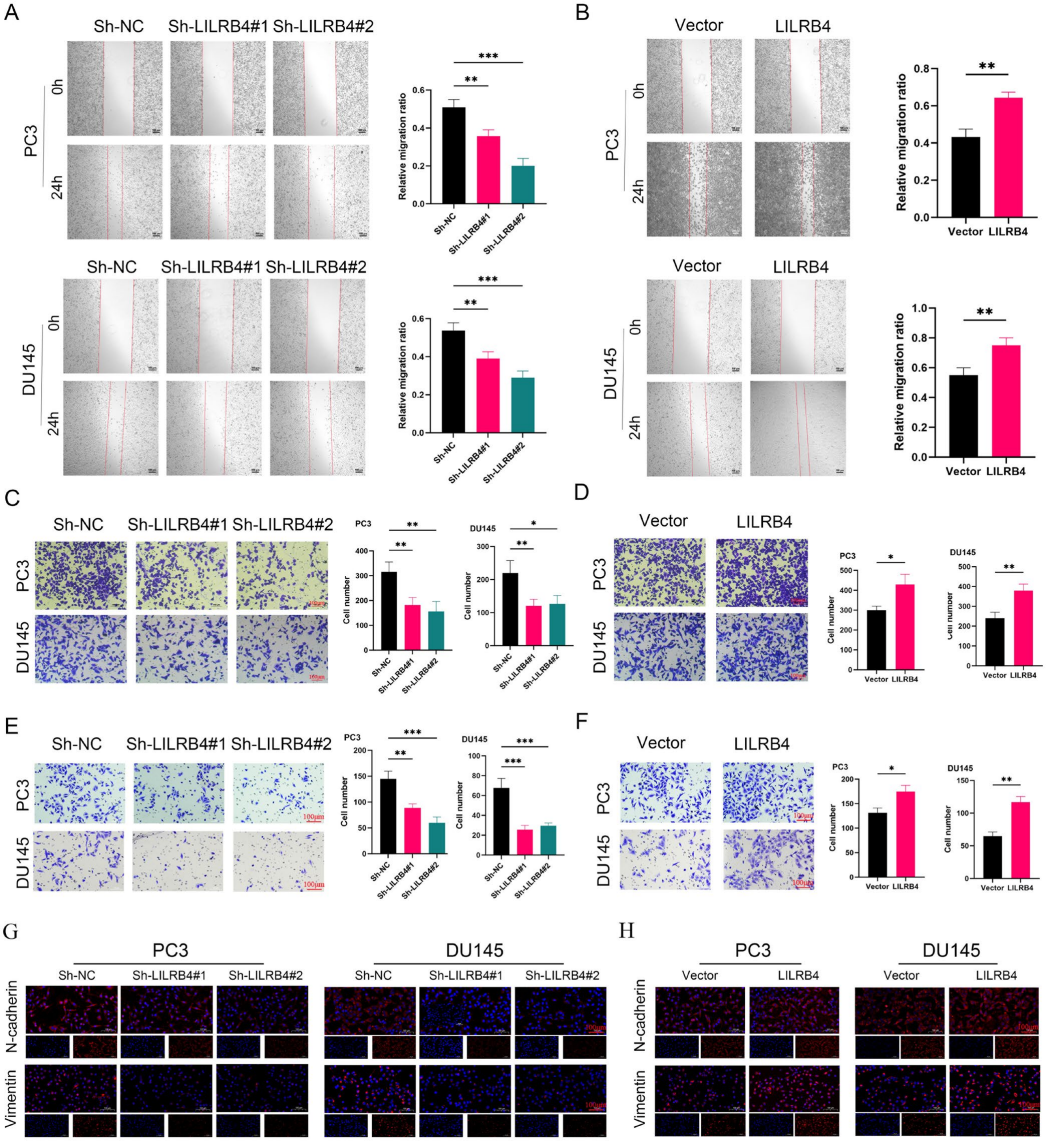
LILRB4 promotes PCa cells migration and invasion. The wound healing assay was conducted to evaluate the effect of migration after LILRB4 silencing (A) and up‐regulation (B). The Transwell migration assay was used after LILRB4 silencing (C) and up‐regulation (D). The Transwell invasion assay was used after LILRB4 silencing (E) and up‐regulation (F). The EMT‐related proteins N‐cadherin and Vimentin expression were photographed by fluorescence microscopy after LILRB4 silencing (G) and up‐regulation (H). *p < 0.05, **p < 0.01, ***p < 0.001.

The impact of LILRB4 on the migratory capacity of PCa cells was further explored. Knockdown of LILRB4 significantly inhibited the healing of cell scratches, indicating reduced migratory ability (Figure 8A). Conversely, overexpression of LILRB4 enhanced the migratory capacity of PCa cells (Figure 8B). Additionally, the number of migrated cells decreased in the sh‐LILRB4 group and increased in the LILRB4 overexpression group (Figure 8C,D). Results from the Transwell invasion assay were consistent with the migration assay (Figure 8E,F). EMT plays a critical role in tumour progression, enhancing invasiveness, promoting metastasis, conferring stem cell‐like properties and altering the tumour microenvironment to support growth [42, 43]. IF staining was employed to investigate the effect of LILRB4 on EMT. The results showed that down‐regulation of LILRB4 led to decreased expression of EMT‐related proteins, N‐cadherin and Vimentin (Figure 8G). In contrast, LILRB4 overexpression had the opposite effect, increasing the expression of these proteins (Figure 8H).

### 4.9 LILRB4 Promotes PCa Progression via NF‐κB and Pi3K/AKT Pathways

**FIGURE 9:**
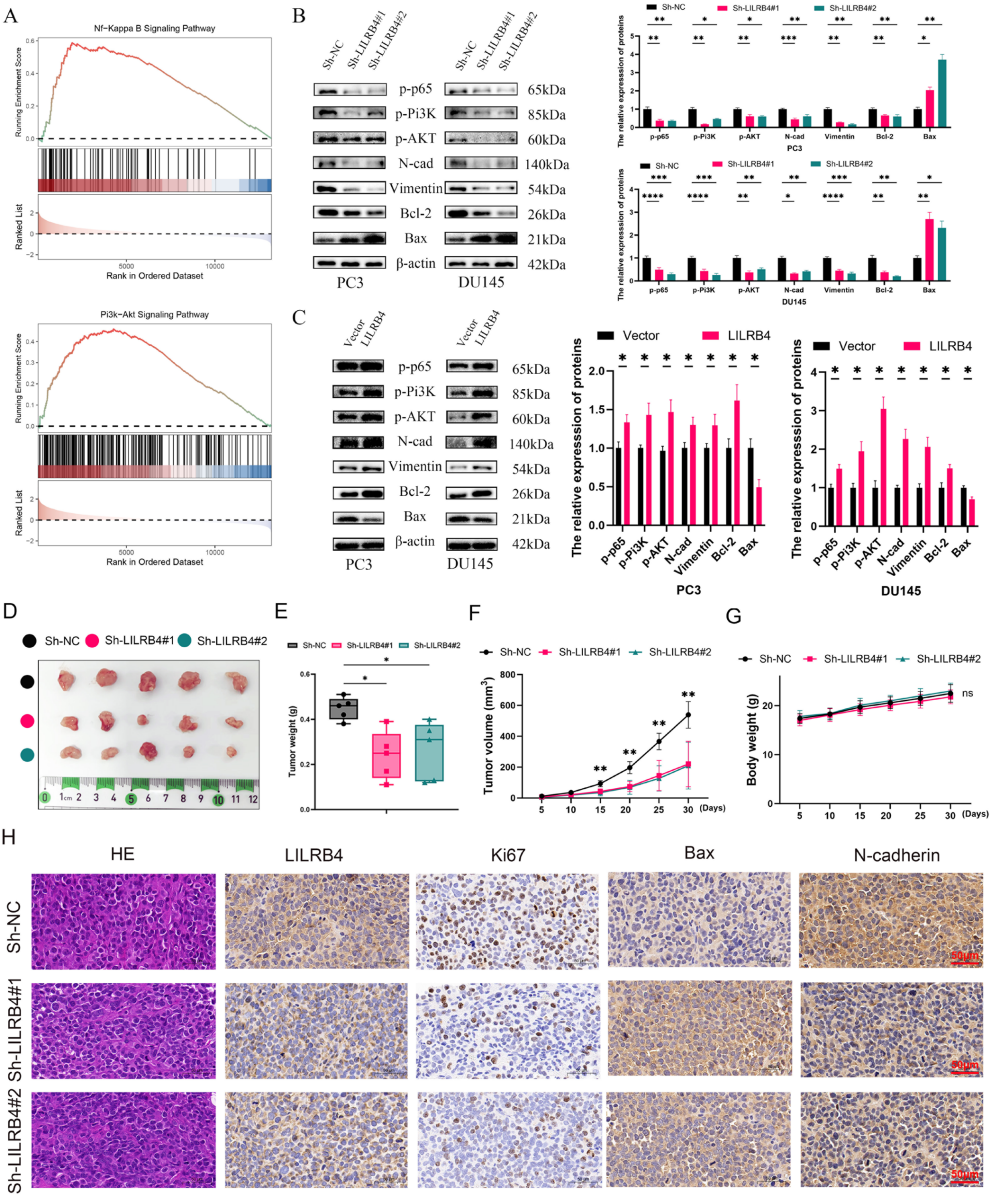
LILRB4 promotes PCa progression via NF‐κB and Pi3K/AKT pathways. (A) The GSEA analysis demonstrated the potential pathways for LILRB4 to promote PCa progression. (B) The effects of silencing LILRB4 on NF‐κB, Pi3K/AKT, EMT and cell apoptosis. (C) The effects of up‐regulation of LILRB4 on NF‐κB, Pi3K/AKT, EMT and cell apoptosis. (D) All the tumours after sacrifice. Comparison of the tumour weight (E), volume (F) and the mice growth curves (G) among three groups. (H) All images of HE and IHC staining of xenografts were shown. *p < 0.05, **p < 0.01, ***p < 0.001, ****p < 0.0001.

To elucidate the mechanisms by which LILRB4 influences PCa, GSEA was conducted. LILRB4 exhibited a positive correlation with the PI3K/AKT and NF‐κB signalling pathways (Figure 9A). Western blot analysis confirmed that LILRB4 knockdown significantly reduced phosphorylation of PI3K/AKT and P65 (Figure 9B), while LILRB4 overexpression had the opposite effect (Figure 9C). Moreover, LILRB4 knockdown in PCa cells led to an increase in the pro‐apoptotic protein Bax and a decrease in the anti‐apoptotic protein Bcl‐2 (Figure 9B,C). The expression of N‐cadherin and Vimentin followed a pattern consistent with LILRB4 expression changes (Figure 9B,C).

To investigate the in vivo role of LILRB4, a xenograft model was established. PC3 cells were injected into the flanks of BALB/c nude mice and tumour growth was measured every 5 days. All tumours were excised on the 35th day (Figure 9D). The xenograft model confirmed that LILRB4 inhibition significantly reduced tumour weight (Figure 9E) and slowed tumour growth (Figure 9F), with no significant change in the mice's body weight (Figure 9G). Additionally, the proliferation marker Ki67 was markedly reduced in the LILRB4 depletion group (Figure 9H) and LILRB4 knockdown also led to increased apoptosis, as indicated by up‐regulation of Bax (Figure 9H). To assess whether LILRB4 regulates EMT in vivo, the expression of N‐cadherin was measured and a significant decrease in N‐cadherin levels was observed upon LILRB4 silencing (Figure 9H). Collectively, these results further support the conclusion that LILRB4 promotes PCa progression via the NF‐κB and PI3K/AKT signalling pathways, positioning LILRB4 as a promising therapeutic target for PCa.

## 5 Discussion

In 1925, Warburg discovered that tumour cells exhibit a dramatic increase in glucose uptake even under aerobic conditions, coupled with the excessive production of lactate, a phenomenon now known as the Warburg effect [44]. Several genes and proteins, such as HIF‐1 and LDHA, are implicated in the glycolytic process in cancer cells [40, 41, 45]. Lactate not only serves as a critical nutrient for cancer cells, but also contributes to tumour growth, metastasis and immune evasion [9, 46]. When lactate accumulates in cells, it modifies proteins through covalent binding, a process known as lactylation [13]. Lactylation plays a complex and significant role in tumours [16, 17, 18, 47, 48], regulating gene expression and protein functions that promote tumour cell growth, epigenetic regulation and drug resistance [14]. PCa is a highly complex disease, exhibiting diverse morphological and clinical phenotypes [1]. Developing an efficient prognostic model is essential for understanding the heterogeneous biological and clinical characteristics inherent to PCa. Recent studies consistently indicate that aberrant lactylation is closely linked to tumorigenesis and PCa progression [47, 49, 50, 51]. However, the role of lactylation related proteins in predicting prognosis and immune regulation within the PCa microenvironment remains unclear.

This study identified two distinct risk sub‐types defined by a set of 18 LRGs, with survival analysis revealing that 13 of these genes significantly influenced PCa prognosis. Patients were then grouped based on their lactylation gene expression patterns, with distinct immune microenvironment landscapes observed between the clusters. These findings suggest that understanding the immune regulatory mechanisms of lactylation in PCa could provide new insights for PCa immunotherapy. Multiple machine learning algorithms and their combinations were employed to construct prognostic models, which demonstrated superior performance compared to traditional methods. The models showed good stability and generalisability through rigorous internal and external validation, with potential applications in predicting patient survival risk and assisting clinicians in developing personalised treatment strategies. Additionally, high‐risk patients exhibited higher TIDE and escape scores, suggesting that their tumour cells were more likely to evade immune system control, thus facilitating tumour proliferation and metastasis and leading to poorer outcomes in immunotherapy. In contrast, the low‐risk group showed higher MSI, indicating a higher mutational burden and potentially stronger immune responses.

Among the three genes identified through machine learning—*LILRB4*, *FMNL3* and *CD53—*only *LILRB4* showed elevated expression in PCa. Consequently, *LILRB4* was selected as the target gene for further investigation. Bioinformatics analysis revealed a positive correlation between LILRB4 expression and that of HIF1A and LDHA, which was confirmed by IHC of PCa tissues. This finding was consistent with lactylation levels, as *LILRB4* expression was notably increased upon treatment with varying concentrations of lactate in PC3 and DU145 cells.

*LILRB4*, an immune checkpoint, plays a pivotal role in the development and immune escape of various cancers [52]. In multiple myeloma, *LILRB4* activates the STAT3‐PFKFB1 pathway to promote cancer cell proliferation [53]. It also contributes to bone lesions in multiple myeloma by enhancing osteoclast differentiation and maturation [54]. Additionally, *LILRB4* facilitates tumour metastasis by modulating myeloid‐derived suppressor cells, adjusting M2 polarisation and inhibiting the miR‐1 family [55]. In lung cancer, LILRB4 directly activates ERK1/2 signalling, which mediates EMT and promotes tumour angiogenesis through recruitment of SHP2 and SHIP1 [19]. As an immune checkpoint, LILRB4 deletion impedes acute myeloid leukaemia (AML) development by reversing the immunosuppressive microenvironment [20]. The intracellular domain of LILRB4 suppresses T‐cell migration and AML cell migration [22]. Furthermore, LILRB4 blockade increases tumour immune infiltrates and reduces exhausted CD8^+^ T cells in solid tumours [56]. Consequently, LILRB4 represents a promising therapeutic target for future immunotherapy. Strategies targeting LILRB4 may reshape the immune microenvironment, enhance T‐cell infiltration and function and offer new treatment options for patients with PCa resistant to current immunotherapies. The machine learning based prognostic model incorporating LILRB4 has significant clinical potential for predicting immunotherapy efficacy in PCa. Combining this model with existing clinical indicators will aid in developing a more precise personalised treatment decision‐making system.

In this study, high‐ and low‐expression models of LILRB4 were constructed in PCa cells to explore its role in PCa progression. Silencing LILRB4 significantly inhibited PCa cell growth, reduced cell metastasis and promoted cell apoptosis, while overexpressing LILRB4 had the opposite effect. To better understand the mechanisms by which LILRB4 regulates PCa progression, GSEA was used to identify relevant signalling pathways. GSEA results showed that LILRB4 was positively associated with the PI3K/AKT and NF‐κB signalling pathways. LILRB4 promotes tumour progression by activating the NF‐κB pathway in multiple myeloma and lung adenocarcinoma [54, 57, 58]. Moreover, LILRB4 may contribute to chronic lymphocytic leukaemia progression by regulating the AKT pathway [59]. Western blot analysis confirmed that silencing LILRB4 significantly reduced the phosphorylation levels of PI3K/AKT and P65. Conversely, overexpression of LILRB4 had the opposite effect. To further validate the in vivo role of LILRB4, a subcutaneous xenograft model in nude mice was conducted. Results from the xenograft model corroborated our hypothesis that inhibiting LILRB4 substantially reduced tumour weight and slowed xenograft growth, with no observed difference in body weight in the mice.

The results indicated that patients grouped according to distinct lactylation gene expression patterns displayed significantly different immune microenvironment profiles, suggesting that elucidating the immune regulatory mechanisms of lactylation in PCa could offer valuable insights for immunotherapy. The machine learning model developed in this study demonstrated potential for predicting patient survival risks. The LRG LILRB4 emerged as a promising biomarker for PCa prognosis, promoting disease progression through the regulation of the NF‐κB and PI3K/AKT pathways.

## 6 Conclusion

This study identified 18 LRGs associated with PCa prognosis. Patients with PCa were classified into two clusters and a prognostic model was constructed based on these genes, enabling accurate prediction of clinical prognosis, immunotherapy response and drug resistance. The machine learning model was validated in both test and validation sets, confirming its potential to predict patient survival risks. Lactate activation of LILRB4 promoted PCa progression by modulating the NF‐κB and PI3K/AKT pathways. In summary, research into lactylation is opening new avenues for the diagnosis, understanding and treatment of PCa.

## Author Contributions

**Qinghua Wang:** formal analysis (equal), validation (equal), writing – original draft (equal). **Xin Qin:** software (equal), validation (equal), visualization (equal). **Yan Zhao:** formal analysis (equal), software (equal). **Wei Jiang:** data curation (equal). **Mingming Xu:** data curation (equal). **Xilei Li:** visualization (equal). **Haopeng Li:** conceptualization (equal), investigation (equal), writing – review and editing (equal). **Juan Zhou:** conceptualization (equal), investigation (equal), writing – original draft (equal). **Gang Wu:** conceptualization (equal), funding acquisition (equal), project administration (equal), resources (equal), writing – review and editing (equal).

## Ethics Statement

Ethical approval was granted by the Ethics Committee of the Tongji Hospital of Tongji University. The animal experiment was approved and conducted under the supervision of the Institutional Animal Care and Use Ethics Committee of the Tongji Hospital of Tongji University.

## Conflicts of Interest

The authors declare no conflicts of interest.

## Supporting information


**Fig S1** The levels of LILRB4 mRNA after treatment with lactate.

**Appendix S1.** Supporting Information.

## Data Availability

All data generated during this study can be obtained upon reasonable request from the corresponding author.
