# Supplementary material for: Lactylation‐Related Gene LILRB4 Predicts the Prognosis and Immunotherapy of Prostate Cancer Based on Machine Learning
Source: J Cell Mol Med. 2025 Jun 27;29(12):e70669. doi: 10.1111/jcmm.70669 (PMC12203412; doi:10.1111/jcmm.70669)
Supplement: Supplementary file 1 — Fig S1 The levels of LILRB4 mRNA after treatment with lactate. [file JCMM-29-e70669-s002.docx]

**Supplementary Materials and methods**

**Quantitative Real-time PCR**

RNA extraction from PC3 and DU145 cells was performed with the Total RNA Isolation Kit (Vazyme, China). The purified RNA was then reverse-transcribed into cDNA using the ABScript Neo RT Master Mix (Abclonal, China), ensuring genomic DNA elimination. For qPCR amplification, we employed the Bright Cycle Universal SYBR Green qPCR Mix (Abclonal, China). All primers were procured from Sangon Biotech (China). LILRB4 (forword) 5' to 3'-TGAGAAGGCACTGGGAGGTACTG, LILRB4 (reverse) 5' to 3'-TGGAGGAGGAGGAAGAGGAGGAG; β-actin (forword) 5' to 3'-CACCATTGGCAATGAGCGGTTC, β-actin (reverse) 5' to 3'-AGGTCTTTGCGGATGTCCACGT.

**Supplementary Results**

**
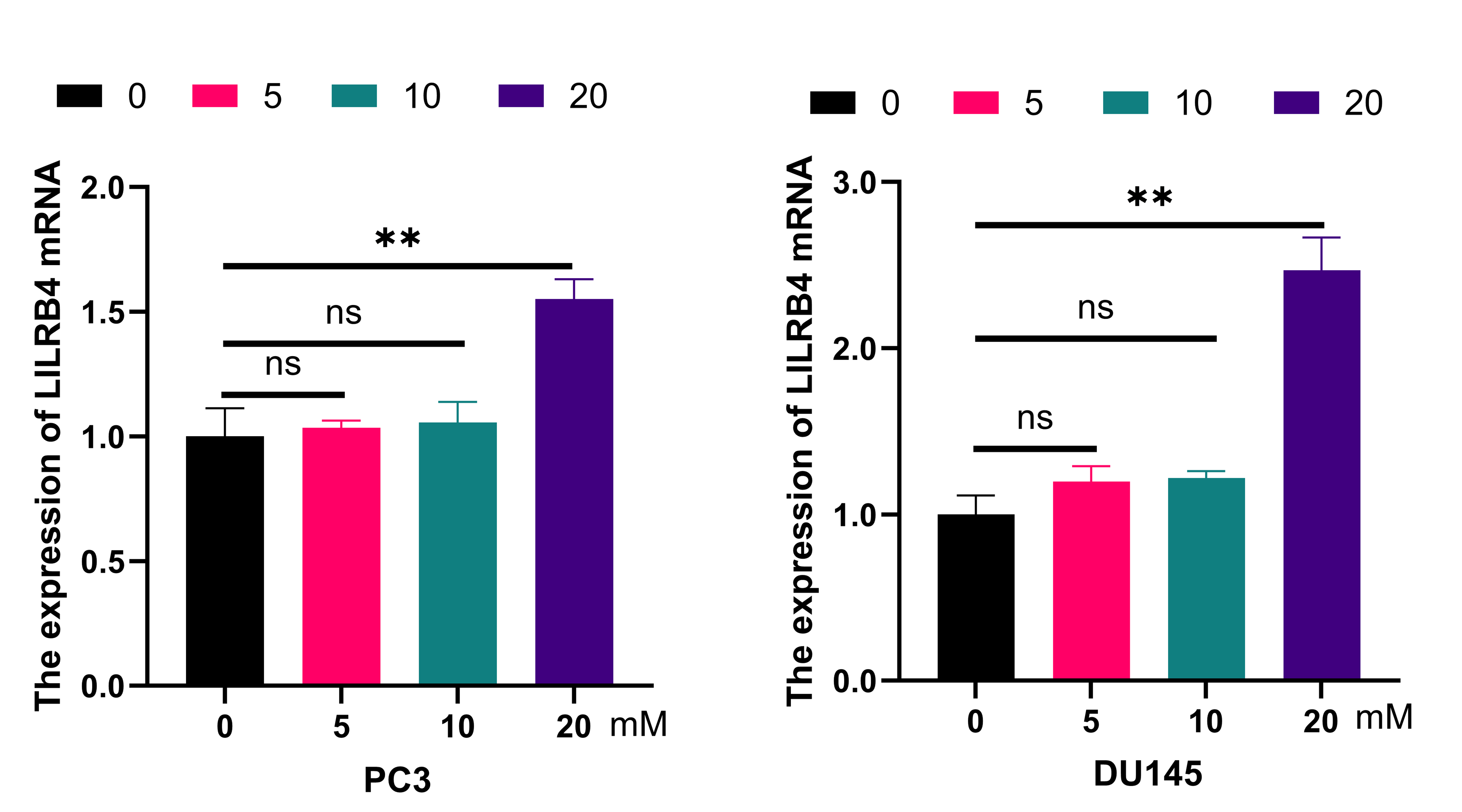
**

**Fig.S1 The levels of LILRB4 mRNA after treatment with Lactate.**
